# Supplementary figures and images for: Identification of Selection Signatures and Loci Associated with Important Economic Traits in Yunan Black and Huainan Pigs
Source: Genes (Basel). 2023 Mar 5;14(3):655. doi: 10.3390/genes14030655 (PMC10048629; doi:10.3390/genes14030655)

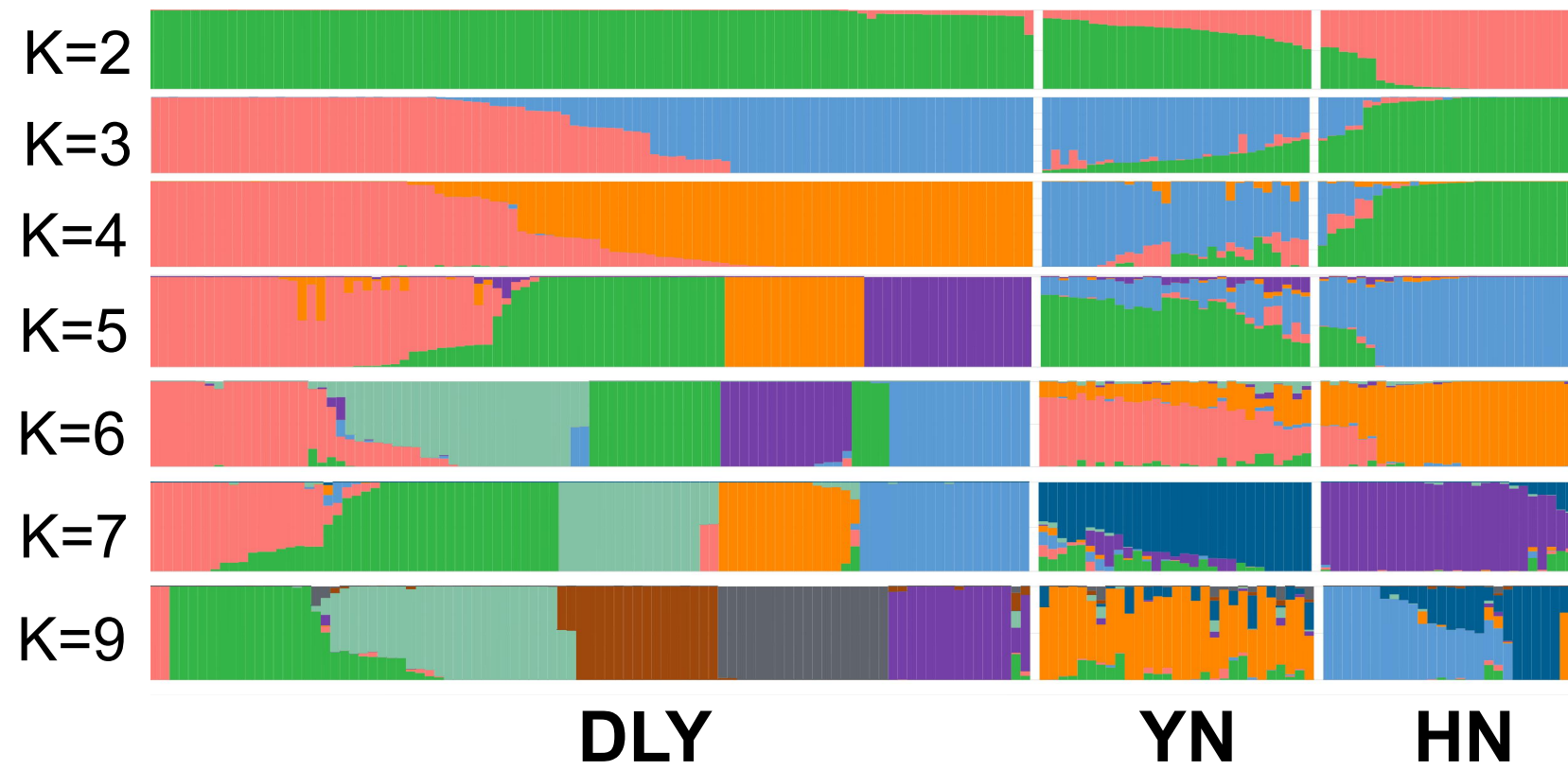

**a****Top 20 of KEGG Enrichment**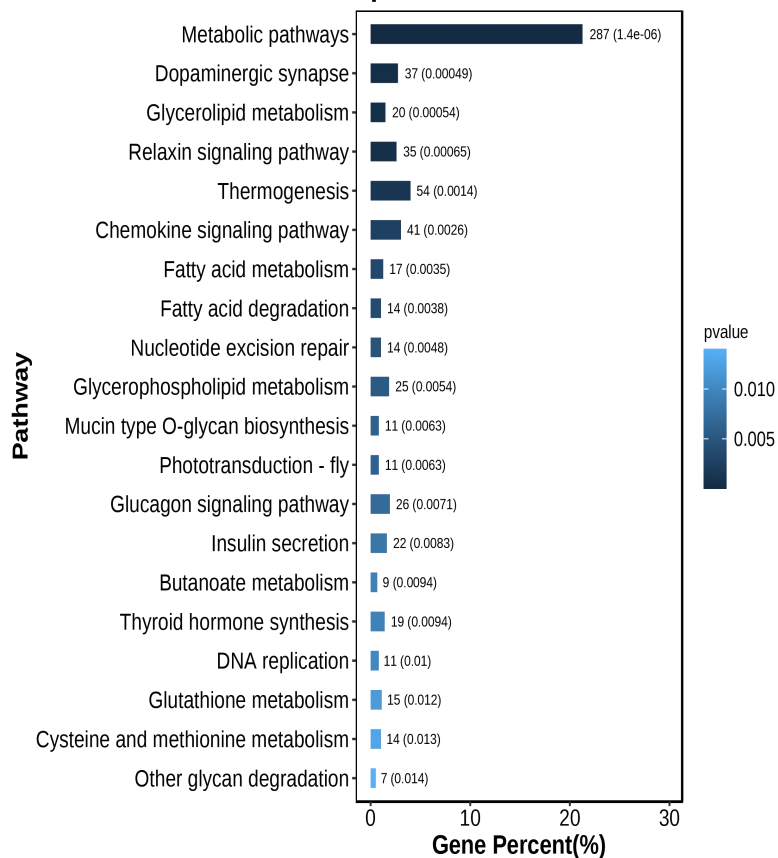**b****Top 20 of KEGG Enrichment**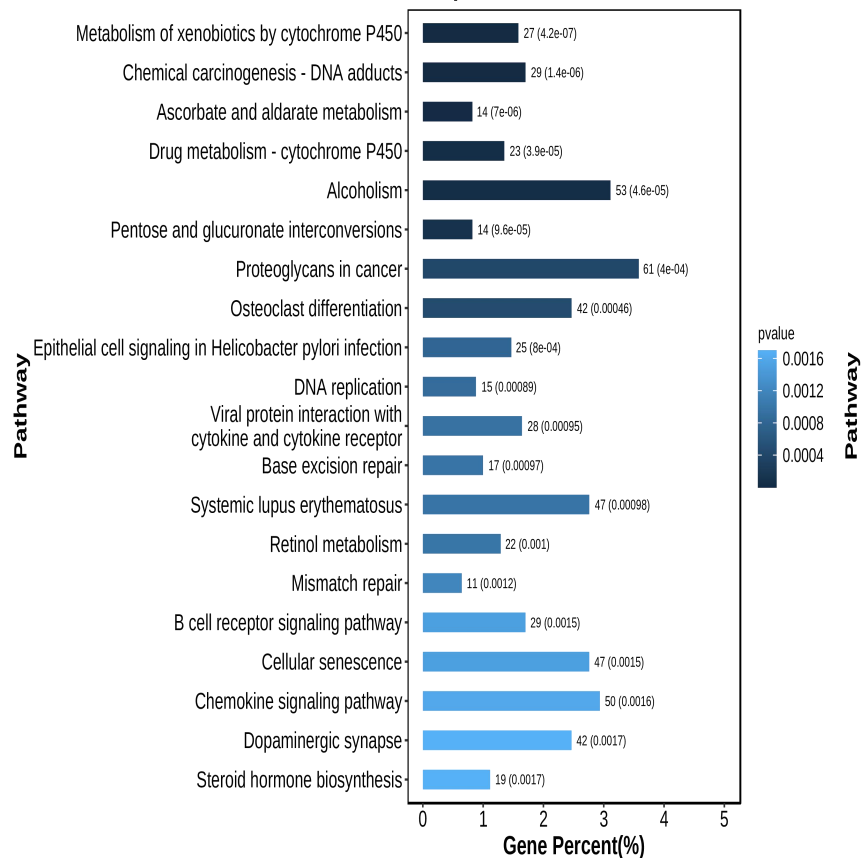**c****Top 20 of KEGG Enrichment**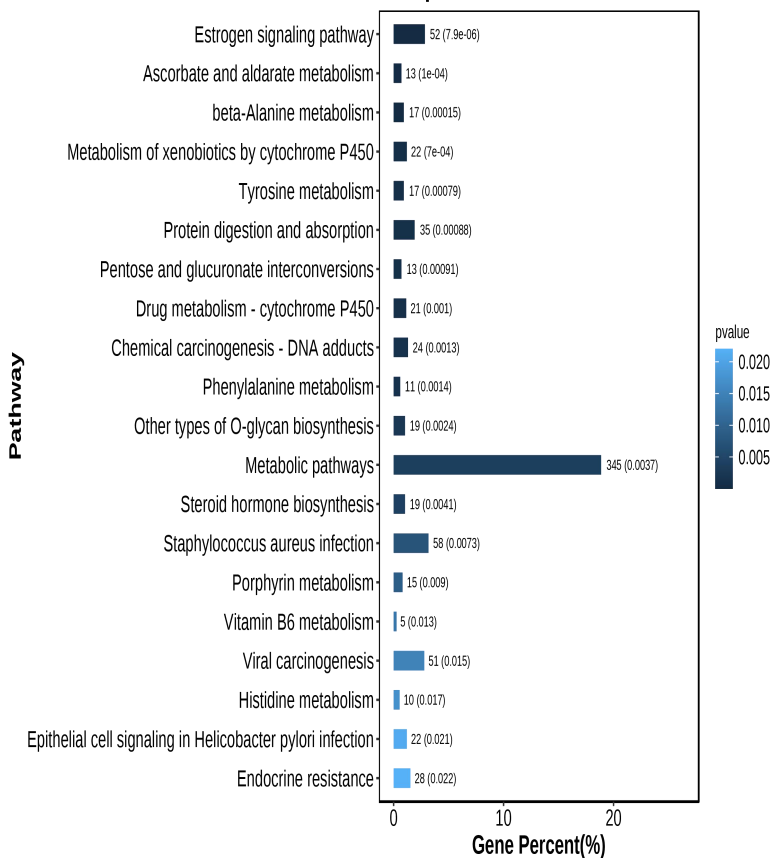

a

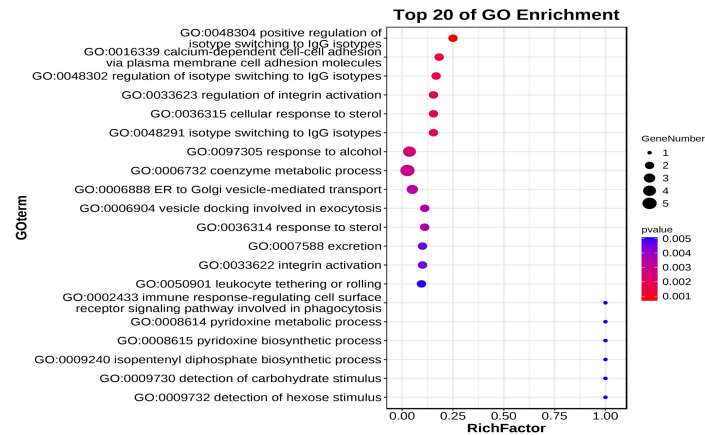

b

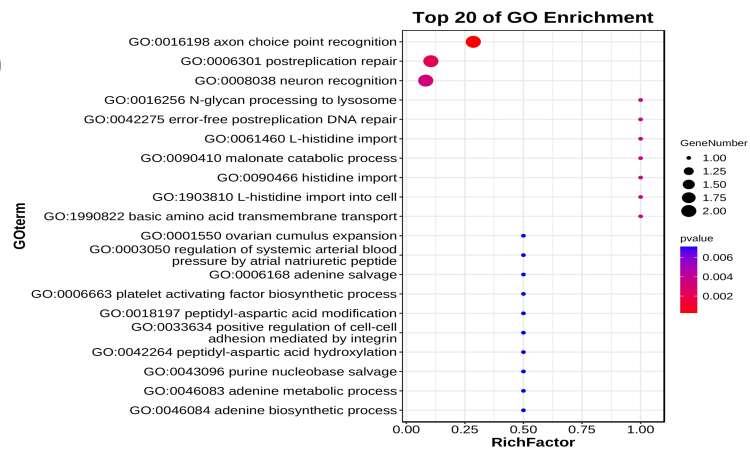

c

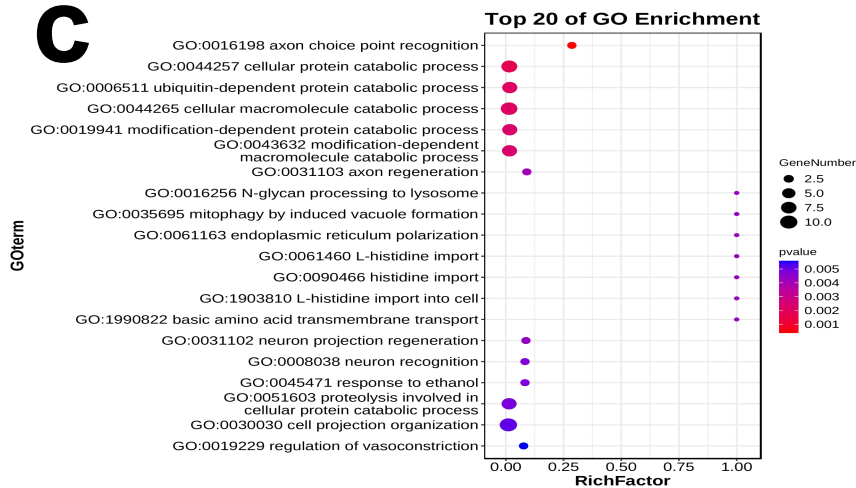

d

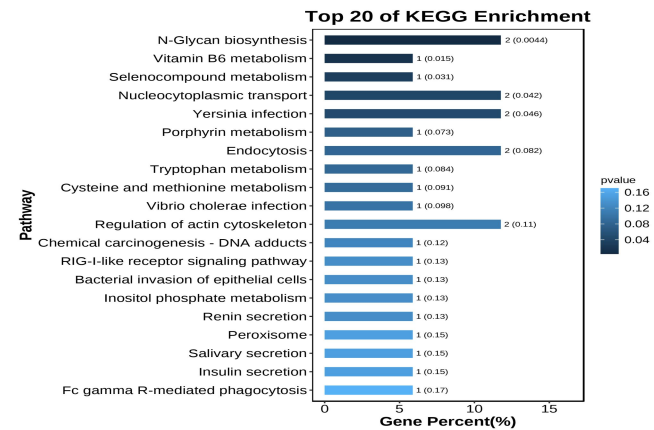

e

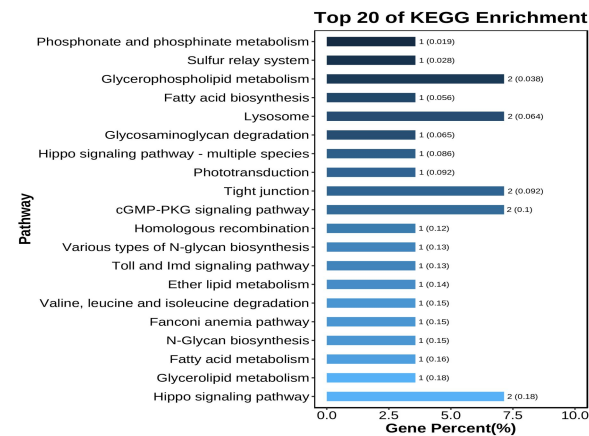

f

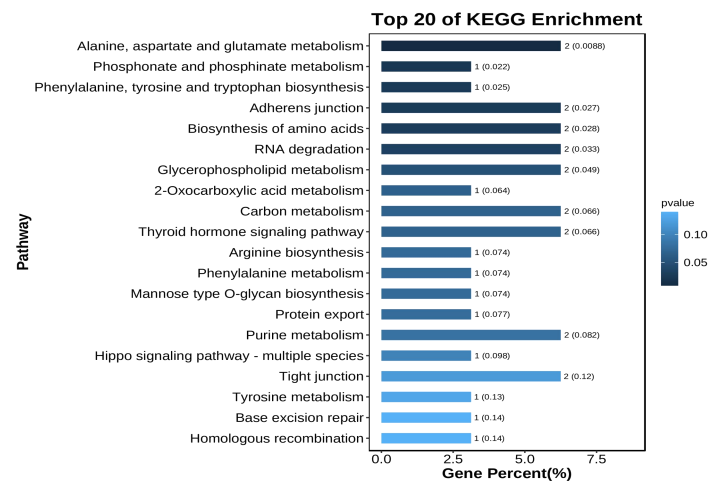

Supplement: Supplementary file 1 [file genes-14-00655-s001.zip › supplementary figures and tables/Figure S1. Population structure of YN black, HN, DLY pigs revealed by admixture analysis with k=2-9..pdf]
